# A multimodal psychological, physiological and behavioural dataset for human emotions in driving tasks

**DOI:** 10.1038/s41597-022-01557-2

**Published:** 2022-08-06

**Authors:** Wenbo Li, Ruichen Tan, Yang Xing, Guofa Li, Shen Li, Guanzhong Zeng, Peizhi Wang, Bingbing Zhang, Xinyu Su, Dawei Pi, Gang Guo, Dongpu Cao

**Affiliations:** 1 College of Mechanical and Vehicle Engineering, Chongqing University, Chongqing, 400044 China; 2 School of Vehicle and Mobility, Tsinghua University, Beijing, 100084 China; 3 Department of Aerospace, Transport, and Manufacturing, Cranfield University, Cranfield, MK430AL UK; 4 Department of Civil Engineering, Tsinghua University, Beijing, 100084 China; 5 School of Mechanical Engineering, Nanjing University of Science and Technology, Nanjing, 210094 China

**Keywords:** Society, Interdisciplinary studies, Human behaviour

## Abstract

Human emotions are integral to daily tasks, and driving is now a typical daily task. Creating a multi-modal human emotion dataset in driving tasks is an essential step in human emotion studies. we conducted three experiments to collect multimodal psychological, physiological and behavioural dataset for human emotions (PPB-Emo). In Experiment I, 27 participants were recruited, the in-depth interview method was employed to explore the driver’s viewpoints on driving scenarios that induce different emotions. For Experiment II, 409 participants were recruited, a questionnaire survey was conducted to obtain driving scenarios information that induces human drivers to produce specific emotions, and the results were used as the basis for selecting video-audio stimulus materials. In Experiment III, 40 participants were recruited, and the psychological data and physiological data, as well as their behavioural data were collected of all participants in 280 times driving tasks. The PPB-Emo dataset will largely support the analysis of human emotion in driving tasks. Moreover, The PPB-Emo dataset will also benefit human emotion research in other daily tasks.

## Abstract (summary)

**Table:** 

Measurement(s)	electroencephalogram measurement • driving behaviour measurement • face expression, body gesture and road scenario measurement • emotion and persionality
Technology Type(s)	electroencephalography (EEG) • driving simulator • visual observation method • self-reported scale

## Background & Summary

Human emotions are integral to daily life, and impacts a variety of cognitive abilities^1,2^. Specifically,they can direct attention to key features of the environment, optimize sensory intake, tune decision making, ready behavioural responses, promote social interaction, and enhance episodic memory^3,4^. Consequently, human emotions affect different tasks that humans experience every day, such as, learning, sleeping, driving, entertainment. Driving is now a typical daily task^5,6^. According to the statistical report, the average driving time per person in the world exceeds 1 hour per day^7,8^, which also is accompanied by serious injuries, fatalities and related costs caused by negative human emotions in driving tasks^9,10^. For a long time, how to reduce accidents risk caused by emotions through studying human emotions in driving tasks has been an important research topic in many fields such as psychology, physiology, engineering, and ergonomics, and has been extensively studied^11–15^.

With the advancement of sensing, machine learning, and computing systems, extensively emerging intelligent vehicles have been developed to connect with vehicles, pedestrians, infrastructures, and clouds in the transportation network^16,17^. Thus, intelligent vehicles have become an intelligent mobile terminal that carries rich functions and services^18,19^, which not only expand and deepen the scope of human-machine interaction but also provide an emerging and challenging research field to further study human emotions in driving tasks, namely, emotion-aware human-machine interactions^20,21^. Specifically, emotion-aware human-machine interactions consists of multi-modal emotion detection and regulation, which will enhance the safety of humans in driving tasks, and will also improve their comfort and driving experience^22,23^. To study the emotion-aware human-machine interactions in driving tasks, cross-disciplinary knowledge is required, including cognitive psychology, brain science, human factors, automotive engineering, and affective computing^19,24^. The latest technological innovations like wearable devices have boosted the study of emotions, leading to a growing number of studies investigating the positive or negative impact of certain emotions during driving (e.g., anger^25^, sadness^26^). In order to measure emotions of human beings, psychological studies have revealed the multi-modal expression of emotion^27^. Further, affective computing studies have focused on the detection of human emotion states based on different emotional expressions^6,28^. Moreover, the human-machine-interface like visual and auditory interface could affect the human’s emotions in driving tasks^15,29^.

To study human emotion in driving tasks, researchers need rich and repeatable datasets^30^. Over the past decade, researchers have shared multiple human emotion datasets in driving. Table 1 summarizes the datasets that capture information of drivers in the surveyed papers. Although these datasets have contributed to successful support in human emotion studies, there is still a lack of multi-modal datasets (including psychological, physiological and behavioural data) that are dedicated to human emotions research in driving. Thus, creating a multi-modal human emotion dataset in driving is an essential step in emotion-aware human-machine interactions studies. However, as far as we know, there is no publicly available multimodal dataset of human emotions in driving tasks.

**Table 1:** The summary of reviewed publicly available datasets for human emotion research in driving.

Dataset	Emotion	Participants	Annotation	Modalities
Ma-dataset^55^	Happy, bothered, concentrated, confused	10	External annotators	Videos (facial expression)
UTDrive DB Classical^56^	Stress (high, low)	77	Driving tasks	Audio, Videos driver), Behavioural (pedal pressure, CAN, GPS)
DriveDB^57^	Stress (high, medium, low)	17	Driving tasks	Physiological data (ECG, EMG, EDA, RESP)
PPB-Emo (Ours)	Anger, sadness, fear, disgust, surprise, happiness, neutral (5 levels, 5 = no emotion, 9 = maximum intensity) Valence, arousal, dominance (9 levels, 1 = not at all, 9 = extremely)	40	Self annotation	Psychological data (Personality), Physiological data (32 channels EEG, 5 types frequency band for each channels), Behavioural data (11 types driving data), Face videos (3 types RGB, 1 type infrared facial expression), Body videos (driver body gesture), Road videos (driving road scenarios)

CAN = controller area network, GPS = global positioning system, ECG = electrocardiography, EMG = electromyography, EDA = electrodermal activity, RESP = respiration, EEG = electroencephalogram.

**Fig. 1:**
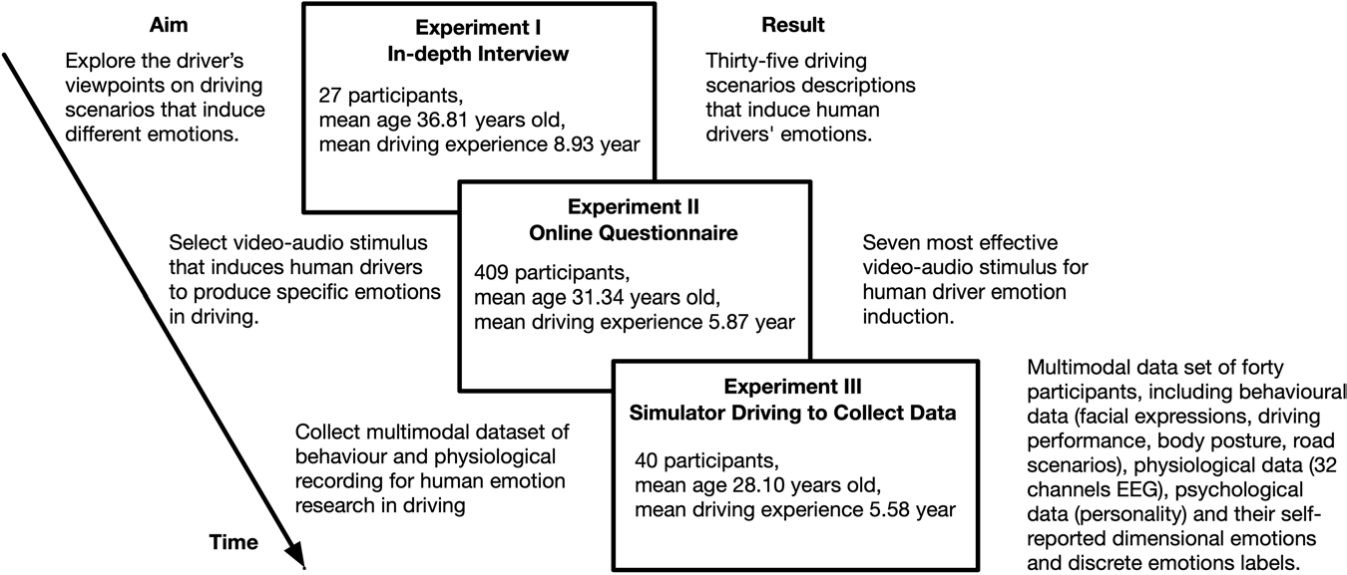
Overview of data collection.

Here, we present the multimodal dataset of psychological, behavioural and physiological data for human emotion (PPB-Emo) in driving tasks. As shown in Fig. 1, we conducted three experiments to collect PPB-Emo dataset. In Experiment I, 27 participants were recruited, the in-depth interview method was employed to explore the driver’s viewpoints on driving scenarios that induce different emotions, and the results were used to develop a questionnaire. For Experiment II, 409 participants were recruited, a questionnaire survey was conducted to obtain driving scenarios information that induces human drivers to produce specific emotions, and the results were used as the basis for selecting video-audio stimulus materials. Experiment III used the video-audio clips selected in Experiment I and Experiment II as the stimulus materials for human driver’s emotion induction. In Experiment III, 40 participants were recruited, and the psychological data (self-reported dimensional emotions and discrete emotions, personality traits) and physiological data (EEG), as well as their behavioural data (driving behaviour, facial expressions, body posture, road scenario) were collected of all participants in 280 times driving tasks. The PPB-Emo dataset will largely support the analysis of human emotion-cognition-behaviour-personality in driving tasks, as well as the study in emotion detection algorithms and adaptive emotion regulation strategies. To the best of our knowledge, The PPB-Emo dataset is currently the only publicly available multimodal dataset of human emotions in driving tasks, and the PPB-Emo dataset will also benefit human emotion research in other daily tasks.

## Methods

### Ethics statement

This study was carried out under the requirements of the Declaration of Helsinki and the later amendments of it. The content and procedures of this study were noticed and approved by the Ethics Committee of Chongqing University Cancer Hospital(Approval number: 2019223).

The written informed consent were given by all participants before they joined in this study. A statement was informed to the participants that results of this study might be published in academic journals or books. During the experiments, participants were told about the rights they would have in experiments. They were allowed to withdraw at any time during the experiments.

The permissions to make the processed data records known to public were gained from all the participants at the end of the study. Since PPB-Emo is to be open to public access, separate consent was obtained for the disclosure of the data that contains personally identifiable information, which is the facial expression of participants during driving tasks. Additional permission was used to inform them about the data types that would be shared in public and the potential risks of re-identification that might be caused by sharing the date and time of the processed data records. The sharing permissions were given by all participants in this study.

### Experiment I: in-depth interview to collect drivers’ viewpoints

Experiment I focused on the investigation of drivers’ viewpoints on driving scenarios that induce different emotions in humans.

#### Participants

In-depth interviews with 27 participants were conducted. The 27 participants included 6 females (22.22%) and 21 males (77.78%). The age range of the participants was 19–55 years old, with an average age of 36.81 years old (standard deviation (SD) = 9.27). Participants’ driving experience ranged from 1 to 25 years, with an average driving experience of 8.93 years(SD = 6.49). The occupations of the participants include workers, teachers, students, farmers, staffs, civil servants, drivers, etc.

#### Procedure

The aim of in-depth interviews was to obtain real-life scenario information that induces different emotions of human drivers and use the results to develop questionnaires. The scenario information collection procedure includes semi-structured interviews with human drivers. The interviews were based on the interview guide method^31^. All participants first signed the demographic questionnaire, and collected personal and demographic information including age, gender, driving experience, and occupation. Then, through interviews with the participants, the participants answered a set of open-ended questions (e.g., question “Could you share an experience that you felt scared while driving or even when you recalled it?”). During the answering process, the interviewer guided the participants to use their own words to recall and describe driving scenarios that trigger different emotions, including roads, weather and lighting conditions; other road users’ behaviours; events; and other contributing factors (e.g., answer “One time when I was driving on a mountain road at night, there was no one on the road. I felt very sleepy. My eyes closed a little uncontrollably. When I opened my eyes, I found that I was in a sharp bend. I stepped on the brakes. It made me feel scared.”). Each participant answered seven driving scenarios questions corresponding to different emotions. The interview time for each participant was about 30 minutes and the process was recorded.

#### Results of collected drivers’ viewpoints

All audio recording and on-site notes of the in-depth interviews were transcribed verbatim and analyzed using Excel files. First, the original transcripts of the 27 interviewees were broken into complete sentences. Next, the two researchers (1 male and 1 female) with expert knowledge and rich experience in drivers’ emotions analysis evaluated sorted the sentences separately and the main scenario information corresponding to the seven emotions in the statement were determined under the consensus of them. After summarizing, there are eleven kinds of scenarios that induce anger in human drivers; sixteen kinds of scenarios that induce happiness in human drivers; ten kinds of scenarios that make human drivers fear; eleven kinds of scenarios that trigger human drivers to feel disgusted; There are ten kinds of scenarios that cause human drivers to feel surprised; Relatively, few scenarios that trigger sadness and neutral of human drivers are five and six respectively. Table 2 summarizes the top five driving scenarios that induce each emotion according to the number of participants.

**Table 2:** Description of the top five driving scenarios that induce each emotion according to the number of participants.

Triggered emotion	Scenario description	Frequency
Anger	The vehicles next always change their way maliciously, or they occupy the road while driving very slow.	12
	The next car decides to cut in line with the turn signal off while the distance between the driver’s car and the car in front is small.	8
	Others keep the high beam on while meeting the car, which affects the vision.	4
	Being in a traffiic jam for a long time.	3
	Being forcibly overtaken.	3
Fear	Driving on a road with no light or pedestrian in the nighttime.	6
	Driving on a mountain road with high cliff beside.	5
	Witnessing some serious traffic accidents while driving.	4
	Feeling very tired after driving for a long time, and even almost causes an accident due to fatigue.	4
	Driving next to a large truck.	4
Disgust	The driver in front keeps throwing garbage, water bottles, and spitting out.	12
	Being forcibly cut in line by a nearby vehicle during a traffic jam.	6
	Seeing a lot of garbage on the road.	5
	Some drivers do not follow the traffic rules.	4
	The passengers in the car is making some uncivilized behaviours.	4
Sadness	Hearing sad things while driving, such as an earthquake.	11
	Witnessing an accident while driving.	10
	Thinking of his/her break-up while driving.	4
	Thinking of his/her family conflicts while driving.	3
	The driver feels sad about his/her poor driving skills when he/she compares himself/herself to other drivers who have good skills.	1
Superise	Seeing some novel things while driving, such as small animals suddenly running out.	16
	Seeing one of his/her acquaintance/friend while driving.	3
	Some unknown problems happened with his/her vehicle, such as some kind of noise.	3
	Seeing some pedestrians walking on the highway.	1
	Suddenly see 100 RMB in the car while driving.	1
Happiness	Noticing interesting things happened on the road and the scenery outside is very beautiful.	16
	Chatting and joking with his/her family and friends while driving and feeling very relaxed.	11
	Driving on a road with few vehicles, especially on highways, and feeling that the whole road belongs to himself/herself.	11
	About to go home or arrive at his/her destination after being busy all day.	8
	Driving while listening to his/her favourite music.	7
Neutral	Driving on all normal town roads.	11
	Encountering slight road congestion.	10
	Driving on a highway.	9
	Driving while listening to soft music.	6
	Driving on a country road.	3

### Experiment II: online questionnaire for stimulus selection

Experiment II focuses on obtaining seven driving scenarios that most effectively induce the corresponding emotions of human drivers through questionnaire surveys, as the basis for the selection of video-audio stimulus materials.

#### Participants

409 Chinese participants were recruited from four countries, including China, the United States, Canada, and Singapore. They were asked to complete an online questionnaire, including 146 women (35.61%) and 263 men (64.39%). The age range of the participants is 18–71 years old, and the average age is 31.34 years old (SD = 10.64). Participants’ driving experience ranges from 1–41 years, with an average driving experience of 5.87 years (SD = 6.69).

#### Procedure

Because online surveys can avoid geographical restrictions on data collection, and previous studies have also verified the effectiveness of online tools in assessing driving behaviour^32,33^. Therefore, an online survey was conducted to collect the data in Experiment II. Based on the outcomes of Experiment I, the online questionnaire consists of two parts and a total of ten questions. The first part is the demographic background. There are three questions, including gender, age, and driving experience. The second part is based on the results of Experiment I and developed seven questions for driving scenarios that induce different emotions in human drivers. These questions correspond to seven emotions that need to be investigated. Each question describes five different driving scenarios. These scenario descriptions are derived from the top five more frequently mentioned scenarios in Experiment I. Participants were asked to select the scenarios most likely to induce corresponding emotions from the five scenarios and they can select more than one scenario (up to five) if they want. It takes about 10 minutes to complete the questionnaire.

The professional online survey platform Sojump (www.sojump.com) was used to design and post the questionnaire. Participants’ answers, region, and answer time were automatically recorded. The survey was distributed in the chat groups of social software (WeChat and QQ). To increase the involvement in the survey, participants will receive a reward of five RMB after completing the survey.

#### Results of stimulus selection

**Fig. 2:**
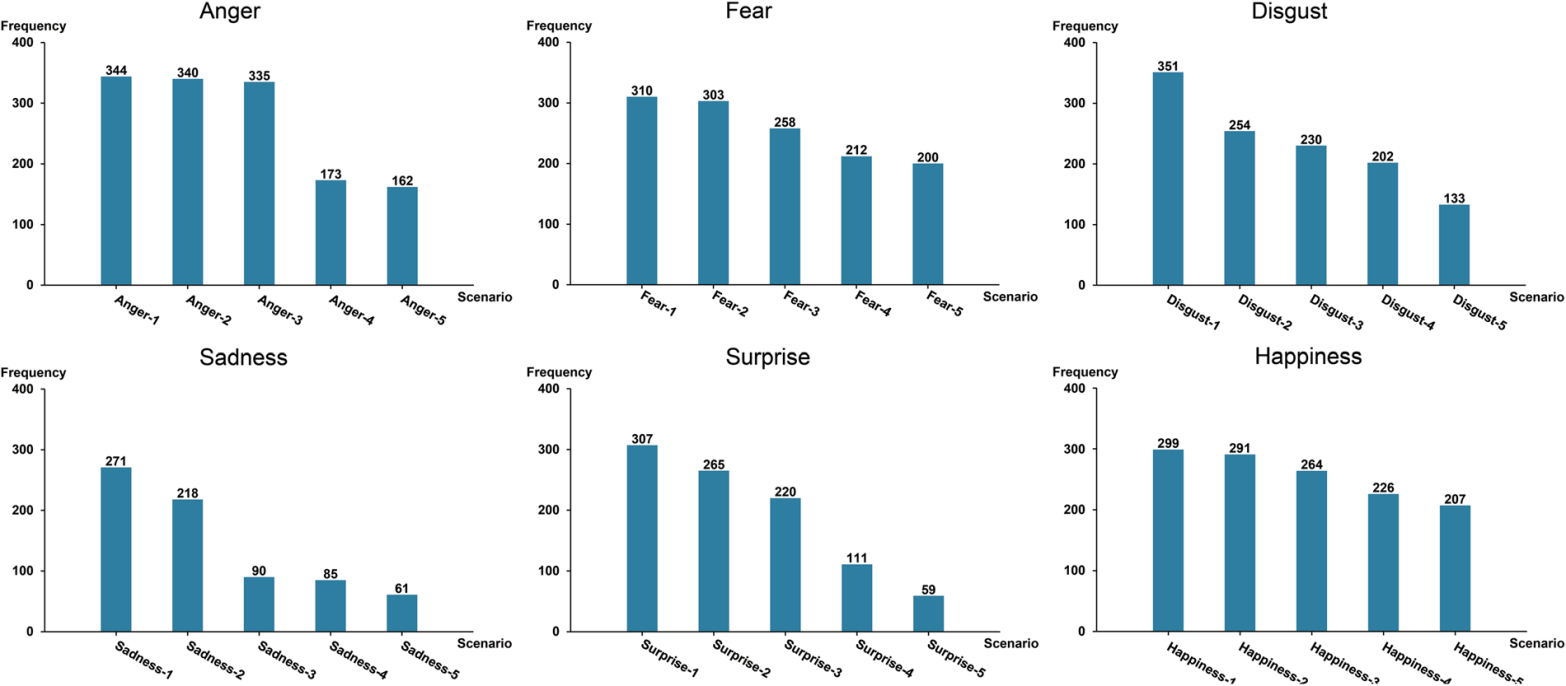
Frequency of the corresponding scenarios that easily induce six basic emotions.The x-axis represents the driving scenarios that trigger a specific emotion, such as anger-1 represents that others keep the high beam on while meeting the car, which affects the vision. Table 3 describes the content of each scenario that triggers a specific emotion. The y-axis shows the frequency of 409 participants’ scenario selections in the online questionnaire and each participant can choose up to 5 scenarios.

Participants reported the corresponding scenarios that easily induce seven kinds of emotion states (anger, fear, disgust, sadness, surprise, happiness and neutral) during driving. Table 3 presents the frequency and percentage of scenarios that easily induce 7 kinds of emotions among the 409 participants. Among them, a total of 344 participants (84.11%) thought that the scenario of “Others keep the high beam on while meeting the car, which affects the vision.” was most likely to induce their anger. 310 participants (75.79%) mentioned “Driving on a mountain road with high cliff beside.” that would make them feel fear. 351 participants (85.82%) felt disgusted when they saw the scenario “The driver in front keeps throwing garbage, water bottles, and spitting out.” A total of 271 participants (66.26%) thought that the scenario of “Witnessing an accident while driving.” was the easiest to make them sad. 307 participants (75.06%) reported that “Seeing some pedestrians walking on the highway.” would make them surprise. Regarding the happiness, 299 participants (73.11%) reported that “Noticing interesting things happened on the road and the scenery outside is very beautiful.” is the easiest to make them happy. The corresponding frequencies of scenarios are shown in Fig. 2. In addition, 273 participants (66.75%) felt neutral when driving while listening to soft music.

**Table 3:** Results of the online questionnaire survey for 409 participants.

Triggered emotion	Scenario	Content description	Frequency	Percentage
Anger	Anger-1	Others Keep the high beam on while meeting the car, which affects the vision.	344	84.11%
	Anger-2	The next car decides to cut in line with the turn signal off while the distance between the driver’s car and the car in front is small.	340	83.13%
	Anger-3	The vehicles next always change their way maliciously, or they occupy the road while driving very slow.	335	81.91%
	Anger-4	Being in a traffiic jam for a long time.	173	42.30%
	Anger-5	Being forcibly overtaken.	162	39.61%
Fear	Fear-1	Driving on a mountain road with high cliff beside.	310	75.79%
	Fear-2	Driving next to a large truck.	303	74.08%
	Fear-3	Feeling very tired after driving for a long time, and even almost causes an accident due to fatigue.	258	63.08%
	Fear-4	Driving on a road with no light or pedestrian in the nighttime.	212	51.83%
	Fear-5	Witnessing some serious traffic accidents while driving.	200	48.90%
Disgust	Disgust-1	The driver in front keeps throwing garbage, water bottles, and spitting out.	351	85.82%
	Disgust-2	Being forcibly cut in line by a nearby vehicle during a traffic jam.	254	62.10%
	Disgust-3	Seeing a lot of garbage on the road.	230	56.23%
	Disgust-4	The passengers in the car is making some uncivilized behaviors.	202	49.39%
	Disgust-5	Some drivers do not follow the traffic rules.	133	32.52%
Sadness	Sadness-1	Witnessing an accident while driving.	271	66.26%
	Sadness-2	Hearing sad things while driving, such as an earthquake.	218	53.30%
	Sadness-3	Thinking of his/her break-up while driving.	90	22.00%
	Sadness-4	Thinking of his/her family conflicts while driving.	85	20.78%
	Sadness-5	The driver feels sad about his/her poor driving skills when he/she compares himself/herself to other drivers who have good skills.	61	14.91%
Surprise	Surprise-1	Seeing some pedestrians walking on the highway.	307	75.06%
	Surprise-2	Some unknown problems happened with his/her vehicle, such as some kind of noise.	265	64.79%
	Surprise-3	Seeing some novel things while driving, such as small animals suddenly running out.	220	53.79%
	Surprise-4	Seeing one of his/her acquaintance/friend while driving.	111	27.14%
	Surprise-5	Suddenly see 100 yuan in the car while driving.	59	14.43%
Happiness	Happiness-1	Noticing interesting things happened on the road and the scenery outside is very beautiful.	299	73.11%
	Happiness-2	Driving while listening to his/her favourite music.	291	71.15%
	Happiness-3	Driving on a road with few vehicles, especially on highways, and feeling that the whole road belongs to himself/herself.	264	64.55%
	Happiness-4	About to go home or arrive at his/her destination after being busy all day.	226	55.26%
	Happiness-5	Chatting and joking with his/her family and friends while driving and feeling very relaxed.	207	50.61%
Neutral	Neutral-1	Driving while listening to soft music.	273	66.75%
	Neutral-2	Driving on a country road.	200	48.90%
	Neutral-3	Driving on a highway.	168	41.08%
	Neutral-4	Driving on all normal town roads.	131	32.03%
	Neutral-5	Encountering slight road congestion.	87	21.27%

The emotions of human drivers need to be induced by appropriate stimuli to collect emotion data. Video-audio clips have been proven to reliably trigger the emotions of human driver^6,34,35^. Based on the results of the questionnaire survey, we manually selected the seven most effective (the highest percentage of participants were selected in each emotional scenario) video-audio clips on the Bilibili website (https://www.bilibili.com/) to induce the corresponding emotions of the human driver in Experiment III. Bilibili is a Chinese video-sharing site where users can upload videos of their lives, and video viewers can tag or add comments to videos through a scrolling commenting system nicknamed “bullet-screen comments”, which will help us evaluate video viewers’ emotional feelings induced by the video-audio clips.

To select the most effective video-audio clips based on the results of the online survey, two research experts (1 male and 1 female) with rich experience in drivers’ emotions analysis evaluated more than 100 video-audio clips. The consensus of the two experts determined the choice of video-audio clips, and finally, 7 videos were selected for Experiment III. Notebaly, in order to make the driver feel more immersive and induce the correct emotion in Experiment III, all the selected video-audio clips in Experiment II were first-perspective of the human driver. Table 4 describes the contents of these seven clips.

**Table 4:** Contents description of the selected seven video-audio stimulus for human driver emotion induction.

Target emotion	Content	Duration(sec)
Anger	Others Keep the high beam on while meeting the car, which affects the vision.	10
Sadness	Witnessing an accident while driving.	15
Disgust	The driver in front keeps throwing garbage, water bottles, and spitting out.	19
Fear	Driving on a mountain road with high cliff beside.	25
Happiness	Noticing interesting things happened on the road and the scenery outside is very beautiful.	28
Neutral	Driving while listening to soft music.	29
Superise	Seeing some pedestrians walking on the highway.	10

### Experiment III: multi-modal human emotion data collection in driving tasks

The aim of Experiment III is to collect the multimodal psychological, physiological and behavioural dataset for human emotions in driving tasks.

#### Participants

A total of 41 drivers from Chongqing were recruited for this data collection experiment. Among these participants, the data of participant 1 was found incomplete and invalid after the collection process. The reason might due to the unexpected technical problems. Therefore, the data of 40 participants (age range = 19–58 years old, average age = 28.10 years old, SD = 9.47)) were valid in this experiment, including 31 males and 9 females. All participants had a valid driver’s license and had at least one year of driving experience (driving experience range = 1–32 years, average driving experience = 5.58 years, SD = 6.02). All participants had normal/corrected vision and hearing. Their health statuses were reported before the start of the experiment. Participants were suggested to have a regular 24-hour schedule and took no stimulating drugs or alcohol before the experiment. Each participants received a reward of 200 RMB after the experiment.

#### Experiment setup

**Fig. 3:**
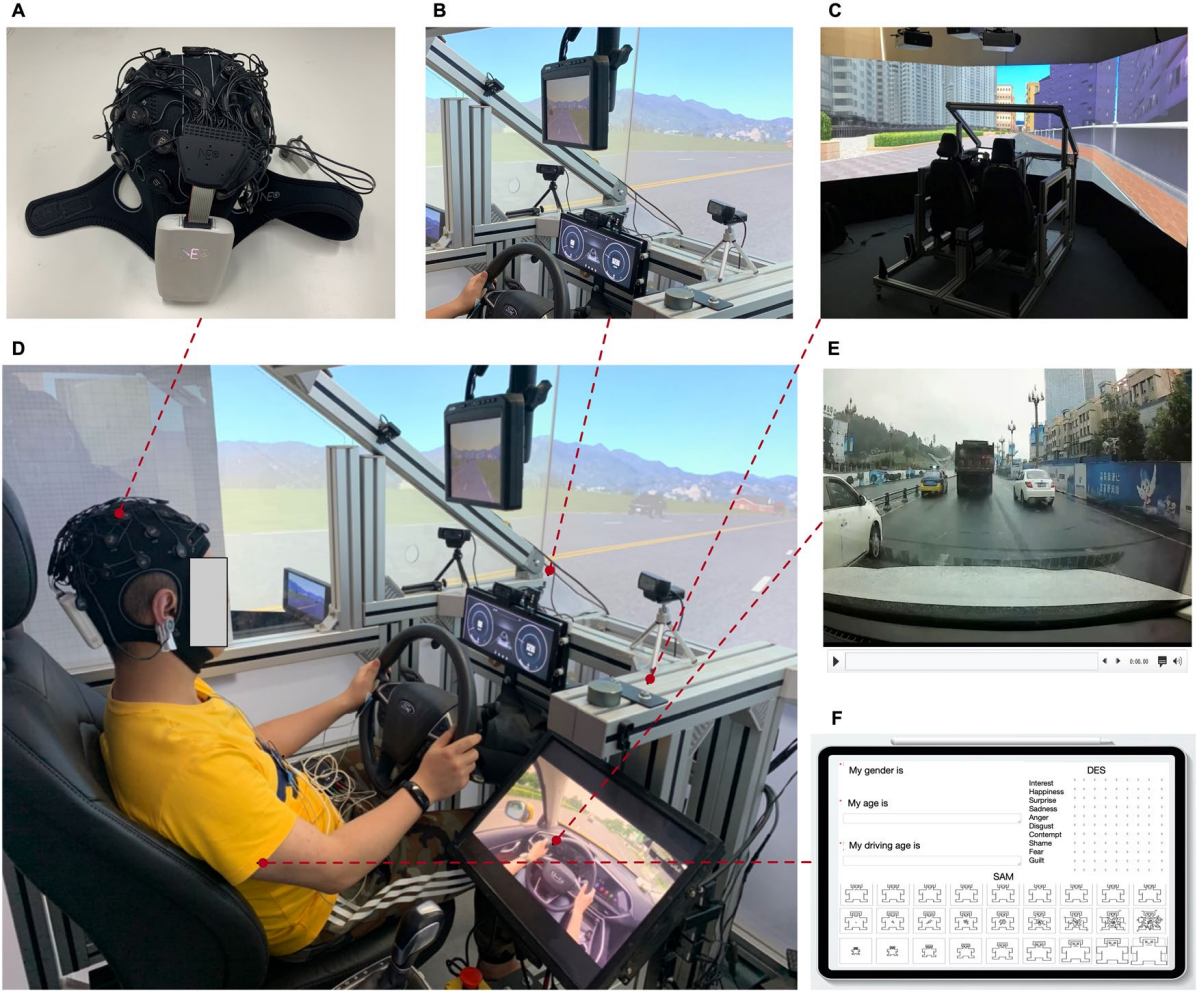
Experimental setup of human driver multi-modal emotional data collection. (**A**) EEG data collection, (**B**) video data collection, (**C**) driving behaviour data collection, (**D**) experiment setup, (**E**) driver’s emotion induction, (**F**) psychological data collection. The use of the relevant portraits in Fig. 3 has been authorized by the participants, and the identifiable information has been anonymized with the knowledge of the participants.

The multi-modal data collection system used in this experiment mainly includes the psychological data collection module, physiological data collection module, behavioural data collection module, driver emotion induction module, driving scenarios, and data synchronization. Figure 3 shows the setup of the overall multi-modal data collection experiment. The contents of the specific modules are as follows:

### Psychological data collection module

In this experiment, three self-reported scales were used to collect psychological data, including self-assessment manikin (SAM), differential emotion scale (DES), Eysenck personality questionnaire (EPQ). SAM^36^ was used for participants to subjectively annotate their dimensional emotions. Representations of non-verbal graphical were used in SAM to evaluate the level of three dimensions (arousal, valence, and dominance). The 9-point scale (1 = “not at all”, 9 = “extremely”) SAM was used for assessment in the experiment procedure. DES^37^ was used for participants to subjectively annotate their discrete emotions. DES is a multidimensional self-report scale for human’s emotions assessment, including ten fundamental emotions: sadness, anger, contempt, fear, shame, interest, joy, surprise, disgust, and guilt. In the experiment, the 9-point scale DES (1 = “not at all”, 9 = “extremely”) was chosen as the method to evaluate the intensity of self-reported emotions in each dimension. EPQ^38^ with a total of 88 questions was used to assess the personality traits of participants in the experiment. EPQ is a multi-dimensional psychological measurement^38^, which can measure the personality traits of humans, including P-Psychoticism/Socialisation, E-Extraversion/Introversion, N-Neuroticism/Stability, L-Lie/Social Desirability. The experiment used iPad (Apple, Cupertino, USA) for participants’ self-reported emotions during driving.

### Physiological data collection module

An EnobioNE (Neuroelectrics, Barcelona, Spain) was used in the experiment to collect participant’s EEG physiological data. EnobioNE is a 32-channel wireless EEG device that uses a neoprene cap to fix the channel at the desired brain location. The electrical activity of the brain was recorded using the EnobioNE-32 system. Dry copper electrodes (coated with a silver layer) fixed on the cap was used to guarantee the good contact with the participant’s scalp. The amplitude resolution of EnobioNE we used was 24 bi (0.05 *μ*V), the sampling rate was 500 Hz, and the band-pass filter was between 2 and 40 Hz. The signal was directly captured by the NIC2 software, and The software contained programs for acquiring and processing signals. During the experiment, the software filtered out electrooculogram (EOG), electromyography (EMG) and electrocardiographic (ECG) signals simultaneously. In addition, the NIC2 software associated the channels with the variable position in the international 10–10 positioning system dynamically. The alpha wave, beta wave, gamma wave, delta wave and theta wave at these positions were directly output to the computer through the NIC2 software.

Before the experiment, the researcher suggested that the participants should wash their hair in advance to avoid the poor contact of the EEG cap electrodes. After the participants put on the device correctly, the contact status of all electrodes in the EnobioNE system was checked and adjusted till a good fit was reached. In addition, a common-mode sensing electrode clamped on the right earlobe was used as a ground reference.

### Behavioural data collection module

Behavioural data collection module consists of driving behaviour data collection and video data collection. Driver behaviour data was obtained using a fixed-based driving simulator (Realtime Technologies, Ann Arbor, USA). The simulator consists of a half-cab platform and an automatic transmission, providing a 270° field of view. The simulator is equipped with a rear-view mirror with a simulated projection, allowing the driver to monitor the traffic behind. Furthermore, the sound of the engine and ambient is emitted through two speakers. The woofer in the simulator simulates the vibration of the vehicle under the driver’s seat. In addition, the simulator dashboard was an LCD (resolution 1920×720, 60 Hz) screen, which was used to display the speedometer, tachometer and gear position. The data of driver behaviour, road information and vehicle posture generated by operating the driving simulator during the driver’s driving process were synchronized and recorded in real-time in the background of the main control computer.

The video data collection composed of six high-definition cameras. Five RGB cameras and one infrared camera were used in this experiment to collect the driver’s face expression, body gesture and road scenario data. The RGB camera we used was Pro Webcam C920 (Logitech, Newark, USA) with a resolution of 1920×1080 pixels, which collected data at a frame rate of 30 fps. The infrared camera we used was an industrial-grade camera with a resolution of 1080×720 pixels, a lens focal length of 2.9 mm and a shooting angle of 90 degrees without distortion. Data collection was at a frame rate of 30 fps. Six cameras were arranged in the cockpit of the driving simulator, of which three RGB cameras were located in front of the participant’s face at 40° on the left and right sides. These cameras were used to collect facial expression data, and one RGB camera was arranged in the front pillar of the driving simulator to collect the driving posture data of participants, and one RGB camera was arranged at the position of the rear-view mirror of the driving simulator to collect road scenario information during driving. Infrared cameras were placed directly in front of the participants’ faces and were also used to collect facial expression data. In addition, the camera was also used to collect the voice information of the participants during emotional driving. The LiveView software (EVtech, Changsha, China) was used to record video information simultaneously from the six high-definition cameras.

### Driver’s emotion induction module

A 20-inch simulator central display (resolution 1280×1024, 60 Hz) was used in the experiment to display video-audio stimulus materials. Stereo Bluetooth speakers (Xiaomi, Shenzhen, China) were used to play audio, and the audio was set to a relatively large volume. Meanwhile, each participant was asked if the volume was comfortable for them to ensure clear hearing volume was adjusted before the experiment. Video-audio stimulus materials selected in Experiment II was used in Experiment III. To ensure that there was no human intervention in the emotion induction of participants during the experiment, the emotion induction system in this experiment was mainly composed of a master computer, a remote display and a remote Bluetooth audio playback device.

### Driving scenarios

In this experiment, two simulated driving scenarios were designed: a formal experimental scenario and a simulated driving practice scenario. The practice scenario setting aims to improve the participants’ control and familiarity with the driving simulator through the practice before the formal experiment. The scenario for practice driving was an 8 km straight section of highway with bidirectional four traffic lanes. The formal experiment scenario is a two-way two-lane straight-line section with a total length of 3 km. The reason for setting these two scenarios is to minimize the requirements of complex driving conditions on the driver’s performance, to show the real multimodal responses elicited by driver emotion to the greatest extent^39^. Participants were asked to drive in the right lane throughout the experiment, keeping the speed at about 80 km/h. The specific configuration parameters of the two experimental scenarios are shown in Table 5. The entire driving scenario uses SimVista and SimCreator software to build the driving scenarios.

**Table 5:** Driving scenarios details of Experiment III.

Driving scenarios	Practice driving			Emotional driving
Length	8 km			3 km
	0~3.2 km	3.2~6.4 km	6.4~8 km	
Speed sign	80 km/h	50 km/h	100 km/h	80 km/h
Oncoming traffic	3 Vehicles/km	6 Vehicles/km	3 Vehicles/km	3 Vehicles/km
Building	4 Buildings/km			2 Buildings/km
Weather	Sunny, high visibility			Sunny, high visibility

### Data synchronization

To collect and store all data synchronously, this experiment used the D-lab data collection synchronization platform (Ergoneers, Gewerbering, Germany) to collect data in multiple channels, including EEG, driving behaviour data, video data are recorded synchronously on a common time axis to achieve subsequent synchronous analysis. In addition, D-Lab was also used to manage and control the experiment.

#### Experiment procedure

**Fig. 4:**
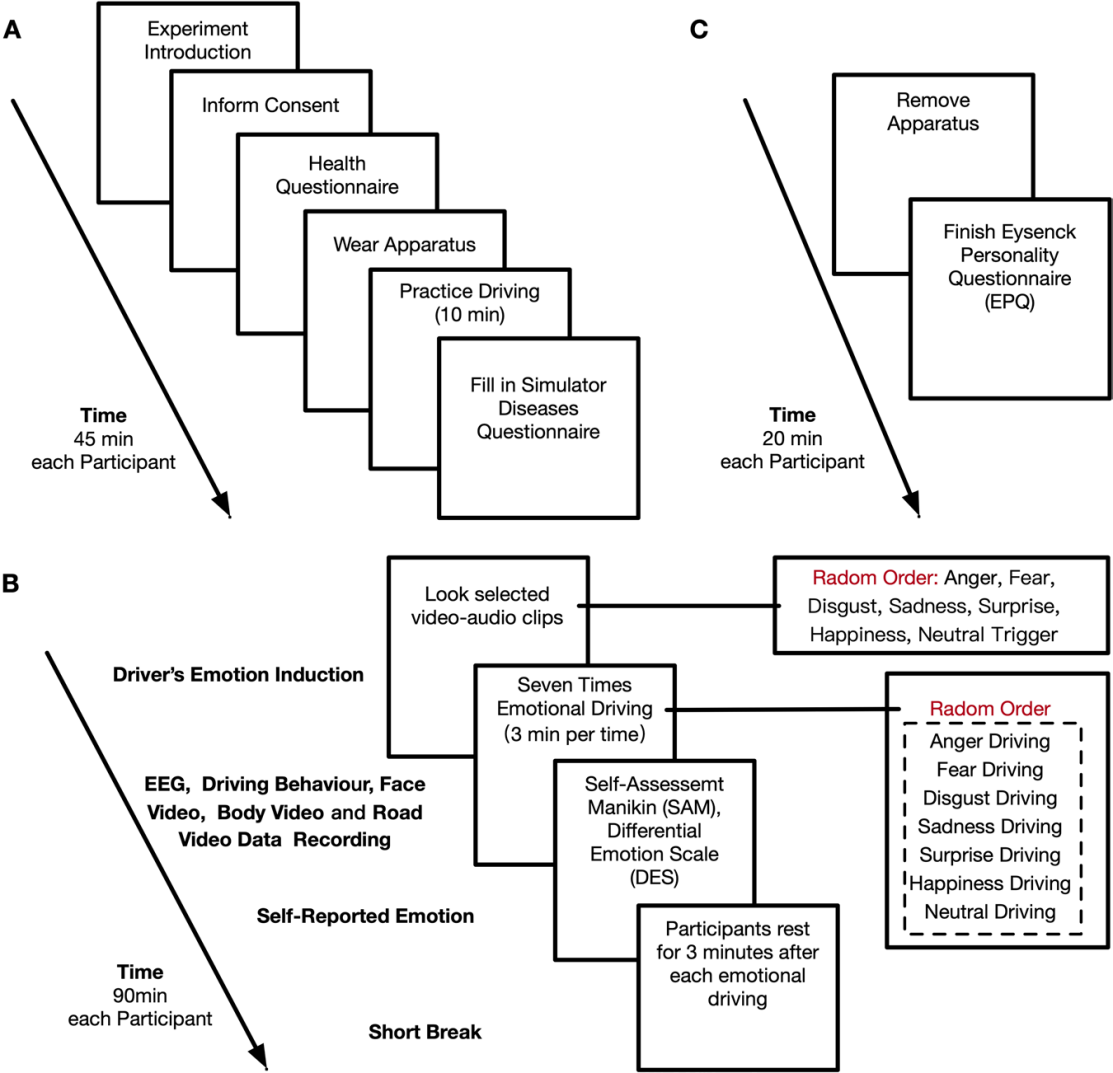
Experimental procedure and tasks of Experiment III. (**A**) Experiment preparation, (**B**) Multimodal human emotion data collection. (**C**) Post-experiment interview.

The whole experiment process is divided into three parts: preparation, emotional driving experiments and post-experiment interviews. The overall process is shown in Fig. 4.

### Experiment preparation

1. Experiment introduction: After the participants arrive in the waiting room, the participants will be explained the purpose, the duration, and the research significance of this experiment. At the same time, the participants will be informed that the data collection apparatus of this experiment is non-invasive and radiation-free, and apparatus will not have any impact or harm on the participants’ health, and the participate voluntarily of participants will be ensured.
2. Sign the participant inform consent form: instruct participants to read the “Participant Inform Consent Form”, the researchers will number the participants and register the basic information.
3. Complete the health form for experiment participants: to check the health of the participants in their daily lives, and whether they have taken psychotropic drugs, cold allergy drugs or alcohol in the past 12 hours. The researchers will evaluate the situation of the participants and see if it is suitable for them to participate in the experiment.
4. Wear testing apparatus: the researchers help the participants to wear the EEG. After wearing the EEG cap, the researchers will adjust the comfort level to observe whether the electrodes are fit and whether the signal collection is normal.
5. Simulator practice driving: the researchers will lead the participants to sit in the cockpit and adjust the positions of the seat to a suitable position. Then, the researchers will help the participants to adapt to the speed control of the driving simulator, and remind participants to drive according to the speed signs. In the process of practice driving, the researchers will explain the formal experiment process, steps and attention to the participants in the co-pilot position.
6. Fill in the driving simulator sickness questionnaire: check whether the participant has any physical discomfort during the driving simulator experiment.

### Multimodal human emotion data collection

In the formal experiment, participants were asked to complete the driving tasks in seven emotional states (anger, sadness, fear, disgust, surprise, happiness, and neutral), in which the order of emotional induction was randomly selected. After each experiment, a 3-minute emotional cooling period was set up to allow participants to calm down from the previous period of emotions.

1. Emotion induction: The researcher loads the preset driving scenarios program to the driving simulator, and at the same time randomly plays the video-audio clips to the participants for emotion induction. The participants watch the video-audio stimulus material and try to maintain their emotions while driving.
2. Emotional driving: After the participant finished watching the emotion induction material, the participant starts emotional driving in D (Driving) gear, and the experimental platform starts recording data simultaneously. Participants were told to keep the speed at around 80 km/h during the emotional driving phase.
3. Self-reported emotion: After completing a time of emotional driving, participants were required to recall the state of their emotions during their driving scenarios by completing self-assessment of the SAM scale and DES scale questionnaires.
4. Repeat the above two steps until the participant completes seven emotional driving. After the participant completes the corresponding SAM scale and DES scale, the researcher will record the experiment process.

### Post-experiment interview

After completing all the emotional driving experiments, the researcher will help the participants to remove the experimental apparatus from their bodies, and then guided the participants to complete the EPQ questionnaire.

## Data Records

### Dataset summary

This section discusses the organisation of PPB-Emo dataset in the Figshare^40^. Table 6 summarizes the data collection of PPB-Emo dataset. After the above data collection, each participant completed 7 simulator driving and data recording. Therefore, for 40 participants, a total of 280 times driving were completed, and the length of each driving was about 135 s. To verify whether the participant experienced the target emotion in certain driving scenario, we carried out the target emotion induction success check.

**Table 6:** PPB-Emo dataset collection summary.

Data collection summary	
Number of participants	40 (9 females, 31 males)
Participants age	19 to 58 (mean = 28.10, standard deviation = 9.47)
Participants driving experience	1 to 32 (mean = 5.58, standard deviation = 6.02)
Stimulus	7 selected video-audio clips
Driving tasks duration	Total 630 min (280sets, about 2.25 min per sets)
Annotations scales	Dimensional emotion model based on SAM
	-Valence
	-Arousal
	-Dominance
	Discrete emotion model based on DES
	-Emotional categories
	-Emotional intensity
Annotations values	Value 1–9
Recorded signals	Participant bio, Personality data, EEG data,
	Driving behavioural data, Facial expression video,
	Driving posture video, Driving scenarios video

The DES of each participant was used as the ground truth to verify whether the target emotion was generated by the participant during the emotional driving. The self-reported emotion would be selected as the ground truth when it was not consistent with the target emotion.The outcomes showed that for each emotional driving, namely anger, sadness, fear, disgust, surprise, happiness and neutral driving, 34, 38, 36, 25, 34, 36 and 37 participants were successfully induced into target emotion, respectively. At the same time, we deleted each set of data that was not successfully induced.

The resulting PPB-Emo dataset contains 240 sets of valid multimodal data from 40 participants, totaling 540 minutes of raw data. It includes psychological data, physiological data and behavioural data of 40 participants during driving tasks. Table 7 summarizes the details of the PPB-Emo dataset dataset.

**Table 7:** PPB-Emo dataset content.

Reocrded data	Content
Emotion induced	Total 240 sets valid multimodal data from 40 participants, including anger (34 sets), sadness (38 sets), fear (36 sets), disgust (25 sets), surprise (34 sets), happiness (36 sets), neutral (37 sets), 30 s per sets
Participant bio	Gender, age, driving age
Personality data	Extraversion, Neuroticism, Psychoticism, Lie
EEG data	Total 120 min, 32 channels EEG data, *α*, *β*, *γ*, *δ*, *θ* data for each channel
Driving behavioural data	Total 120 min, acceleration, lateral-acceleration, gas-pedal-position, brake-pedal-force, gear, steering-wheel-position, velocity, lateral-velocity, x-position, y-position, -position
Facial expression	Total 120 min, including 4 images, central RGB video, left RGB video, fight RGB video, and central infrared video
Driving posture	Total 120 min, participants’ driving behaviour RGB video
Driving scenarios	Total 120 min, road scenarios RGB video
Emotion categories labels	7 categories, anger, sadness, fear, disgust, surprise, happiness and neutral
Emotional intensity labels	5 levels of anger, sadness, fear, disgust, surprise, happiness - 5 = no emotion, 9 = maximum intensity
Valence, arousal, dominance labels	9 levels of valence, arousal, dominance- 1 = not at all, 9 = extremely

### Dataset content

The information in participant-level was pre-processed to accomplish de-identification in accordance with the General Data Protection Regulation (GDPR)^41^. For time synchronization across data, we convert all timestamps from UTC + 8 to UTC + 0 and clipped the raw data. Previous studies have shown that the physiological expression of human emotions can last at least 30 s^15,42^. Therefore, based on the gear change information of the driving behaviour data, we regard the multi-modal data 30 s after the participants start driving as the most effective data in data processing.

For the EEG data and driving behaviour data of each participant, we first exported the raw data from D-Lab, the data format is.txt. Then we converted the data format to.csv, and clipped the first 30 s of driving behaviour and EEG data as the most effective data, renamed and stored them respectively. For the video data of each participant, we first exported the original video data from Liveview, the data format is.mp4. Subsequently, we clipped the first 30 s of the video data as the most effective data, and divided the original shot into 6 images, respectively stored and renamed, including road scenarios video, infrared middle facial video, driving body gesture video, RGB left facial video, RGB middle face video, RGB right face video. Please note that any unedited video and raw log-level data recordings will not be provided. Meanwhile, the code for pre-processing of these data will not be in public either, because the privacy-sensitive information contained exceed the boundaries of the information we are allowed to share. More details can be found in the section of code availability.

In the root path of the dataset, it was organized into the following seven main directories: psychological data, physiological data, driving behavioural data, facial expression data, body gesture data, road scenario data, and scripts. The README.TXT file in these directories will give a detailed explanation. For each participant, a unique two-digit participant ID is assigned.

#### Psychological_data

This directory contains the data of participant biographic, self-reported emotion labels and personality traits as three.xlsx files.

*BIO.XLSX*. Each row contains the biographical data of a participant, organized by participant ID, gender (1 = male, 2 = female), age and driving age.

*Emotion label.XLSX*. Each row contains the self-reported data of a participant’s experienced emotions, organized according to participant ID, valence, arousal, dominance, category, and intensity. The SAM scale was used to measure valence, arousal, and dominance, the DES scale was used to measure category and intensity. The organization of content in each row is shown in the Table 10.

*EPQ.XLSX*. Each row contains the Eysenck personality questionnaire data of a participant, organized according to participant ID, P-score, E-score, N-score, L-score, Where P reï¬‚ects psychoticism/socialisation, E is extraversion/introversion, N means neuroticism/stability, and L is lie/social desirability.

#### Physiological_data

40 sub-folders are further divided in this directory, each sub-folder contains the data of all the EEG signal per participant. These sub-folders were named after the participant ID and include multiple CSV files. Each CSV file corresponds to valid emotional driving. Each row contains the EEG data at an instantaneous measurement and is organized according to rec-time, UTC, 32 channels EEG data, *α, β, γ, δ, θ* frequency bands data for each channel. The organization of content in each row is shown in the Table 8. Besides, the EEG montage description file is contained in the directory. The TXT file described the channels’ information created to display activity over the entire head and to provide lateralizing and localizing information, which will help the understanding and analysis of EEG data.

**Table 8:** Organization of the content in EEG.CSV.

Column	Content	Unit	Sampling rate
1	rec-time	s	250 Hz
2	UTC	s	250 Hz
3–34	*δ* frequency band for 32 channels	Hz	—
35–66	*θ* frequency band for 32 channels	Hz	—
67–98	*α* frequency band for 32 channels	Hz	—
99–130	*β* frequency band for 32 channels	Hz	—
131–162	*γ* frequency band for 32 channels	Hz	—
163–194	EEG data for 32 channels	µV	250 Hz

#### Driving_behavioural_data

40 sub-folders are further divided in this directory, each sub-folder contains all the driving behavioural data (DBD) of one participant. These sub-folders were named after the participant ID and include multiple CSV files. Each CSV file corresponds to valid emotional driving. Each row contains the driving behavioural data at an instantaneous measurement and is organized according to rec-time, UTC, acceleration, lateral-acceleration, gas-pedal-position, brake-pedal-force, gear, steering-wheel-position, velocity, lateral-velocity, x-position, y-position, z-position. The organization of content in each row is shown in the Table 9.

**Table 9:** Organization of the content in driving behavioural data.CSV.

Column	Content	Unit	Sampling rate
1	rec-time	s	50 Hz
2	UTC	s	50 Hz
3	Acceleration	m/s2	50 Hz
4	Lateral acceleration	m/s2	50 Hz
5	Gas pedal position	degree	50 Hz
6	Brake pedal force	N	50 Hz
7	Gear	—	50 Hz
8	Steering wheel position	rad	50 Hz
9	Velocity	m/s	50 Hz
10	Lateral velocity	m/s	50 Hz
11	Vertical velocity	m/s	50 Hz
12	X axis position	m	50 Hz
13	Y axis position	m	50 Hz
14	Z axis position	m	50 Hz

**Table 10:** Organization of the content in emotion labels.CSV, Note: AD = angry driving, SAD = sad driving, FD = fear driving, DD = disgust driving, SD = surprise driving, HD = happy driving, ND = neutral driving.

Column	Content	Value range	
1	Participant ID	2	41
2	Valence	1 = Extremely negative	9 = Extremely negative
3	Arousal	1 = Extremely calm	9 = Extremely excited
4	Dominance	1 = Extremely submissive	9 = Extremely dominant
5	Category	AD, SAD, DD, FD, HD, ND, SD	
6	Intensity	5 = Not at all	9 = Extremely
7	.MP4 file name corresponding to the central RGB facial expression data	—	—
8	.MP4 file name corresponding to the left RGB facial expression data	—	—
9	.MP4 file name corresponding to the right RGB facial expression data	—	—
10	.MP4 file name corresponding to the central infrared facial expression data	—	—
11	.MP4 file name corresponding to the body gesture data	—	—
12	.MP4 file name corresponding to the road scenario data	—	—
13	.CSV file name corresponding to the driving behavioural data	—	—
14	.CSV file name corresponding to the EEG data	—	—

#### Facial_expression_data

**Fig. 5:**
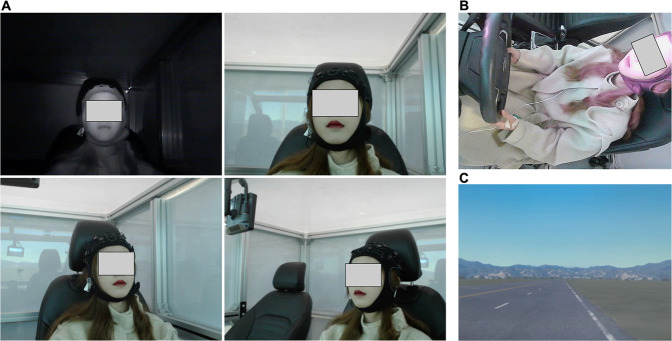
Video data content of PPB-Emo dataset. (**A**) facial expression data, including central infrared facial expression, central RGB facial expression, left RGB facial expression, right RGB facial expression; (**B**) body gesture data, (**C**) road scenario data. The use of the relevant portraits in Fig. 5 has been authorized by the participants, and the identifiable information has been anonymized with the knowledge of the participants.

40 sub-folders are further divided in this directory, each sub-folder contains the data of all the facial expression per participant. The sub-folders are named after the participant ID and include 4 sub-sub folders which are central RGB (CRGB) facial expression data, left RGB (LRGB) facial expression data, right RGB (RRGB) facial expression data, and central infrared (CIR) facial expression data. Each folder contains multiple MP4 files, and each MP4 file corresponds to valid emotional driving. The facial expression data of a participant’s emotional driving record is shown in the Fig. 5.

#### Body_gesture_data

40 sub-folders are further divided in this directory, each sub-folder contains the data of body gesture per participant. These sub-folders were named after the participant ID and include multiple MP4 files. Each MP4 files file corresponds to valid emotional driving. The body gesture data of a participant’s emotional driving record is shown in the Fig. 5.

#### Road_scenario_data

This directory is divided into 40 sub-folders, each of which contains the data of driving road scenario per participant. These sub-folders were named after the participant ID and include multiple MP4 files. Each MP4 files file corresponds to valid emotional driving. The driving road scenario data of a participant’s emotional driving record is shown in the Fig. 5.

#### Selected_stimulus_clips

This directory contains one sub-folder of selected video-audio clips and one clip raw links.xlsx file.

*Selected video-audio clips*. This sub-folder contains seven selected video-audio clips for driver emotion induction. The target emotions corresponding to the seven clips are anger, sadness, disgust, fear, happiness, neutral and surprise.

*Selected video-audio clips raw links.XLSX*. Each row contains the selected video-audio clips raw link of a target emotion, organized by target emotion and raw link.

#### Scripts

The preprocessing and main analysis codes (Python scripts) are summarized in this directory. All results in the technical verification section can be copied using these scripts. For more details, please read the instructions in the README.md file.

## Technical Validation

Our validation consists of reliability validation of emotion labels, quality validation of physiological and behavioural data, as well as correlation analysis of physiology, behaviour and emotion labels.

### Reliability validation of emotion labels

In this section, a K-Means cluster algorithm^43^ was performed to provide a intuitive visualization analysis for the distribution of 40 participants’ subjective rating scores. Then the distribution of each emotion labels was summarized using 3-dimension histograms.

#### Clustering and visualization analysis of emotion labels

**Fig. 6:**
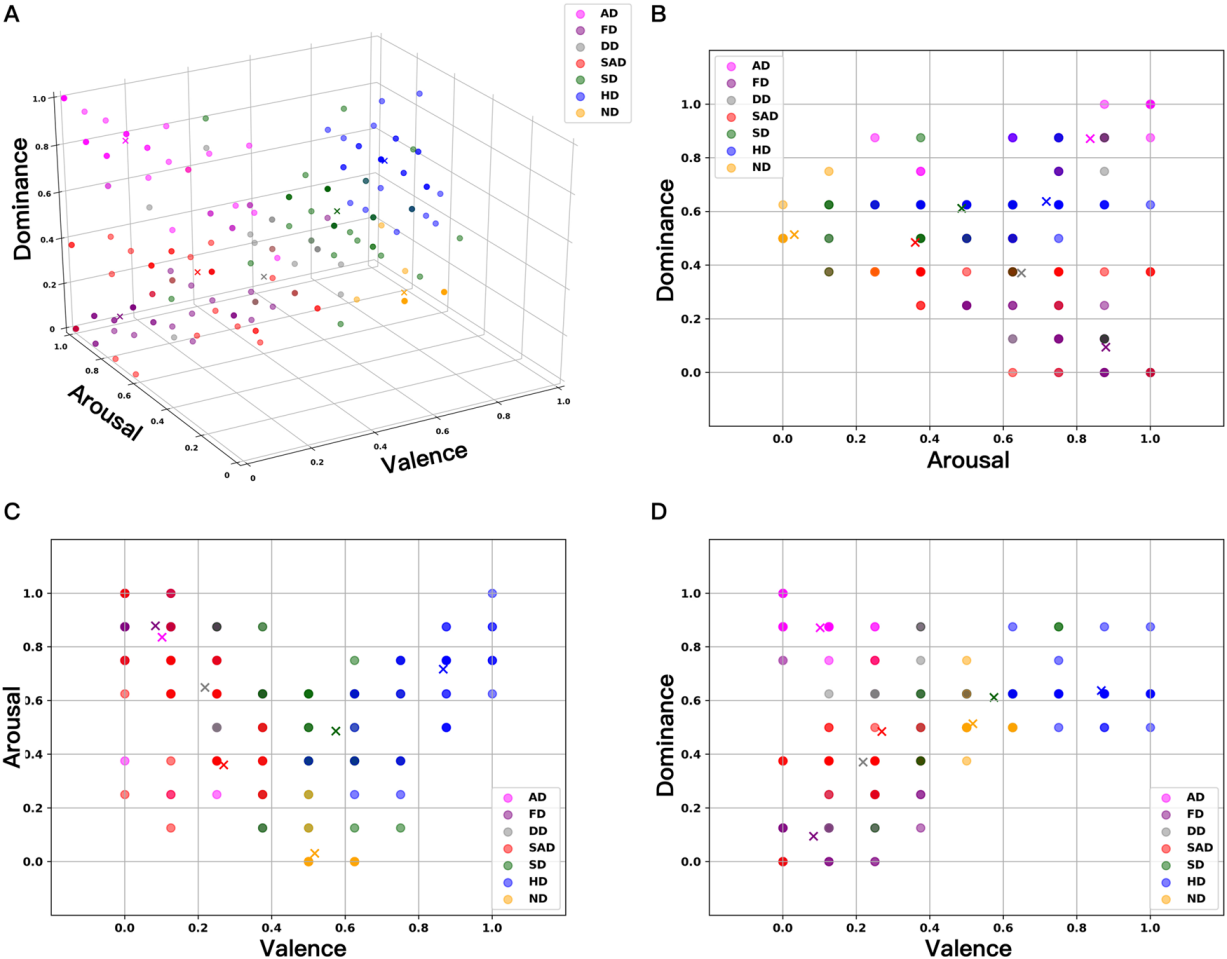
(**A**) shows the full picture of the scatter diagram; (**B**–**D**) shows the distribution of points on each projection surface. AD = angry driving, FD = fear driving, DD = disgusted driving, SAD = sad driving, SD = surprised driving, HD = happy driving, ND = neutral driving.

The SAM we used evaluated emotions in three dimensions: valence, arousal and dominance. To validate the reliability, we performed data clustering and visualization analysis towards this three dimensions. To guarantee that each feature is equally treated, we non-dimensionalized the data by projecting all of the subjective scores to a range of 0 to 1 using the max-min normalization method. Then, the values of valence, arousal, and dominance were used as the coordinate values of the scatter diagram. Figure 6(A) shows the distribution of 40 participants’ rating score after normalization.

The rating scores were clustered using a K-Means algorithm, and the center of each cluster is shown in the Fig. 6(A). Seven clusters represent seven discrete emotion including, anger driving, fear driving, disgust driving, sad driving, surprise driving, happy driving and neutral driving. The center points of each cluster have no overlap and the classification of clusters is relatively obvious, especially the Happy Driving, Angry Driving and Fear Driving scenario. Other clustered points are partially overlapped due to the complexity of emotions and the fact that the participants’ different understanding of certain emotions while they did the scoring. To provide a more comprehensive display, we projected the 3-D scatter diagram into 2-D diagrams. Figure 6(B–D) shows the results after projection, and the center points of each cluster still have no overlap and the classification of clusters is relatively obvious.

#### Distribution analysis of emotion labels

**Fig. 7:**
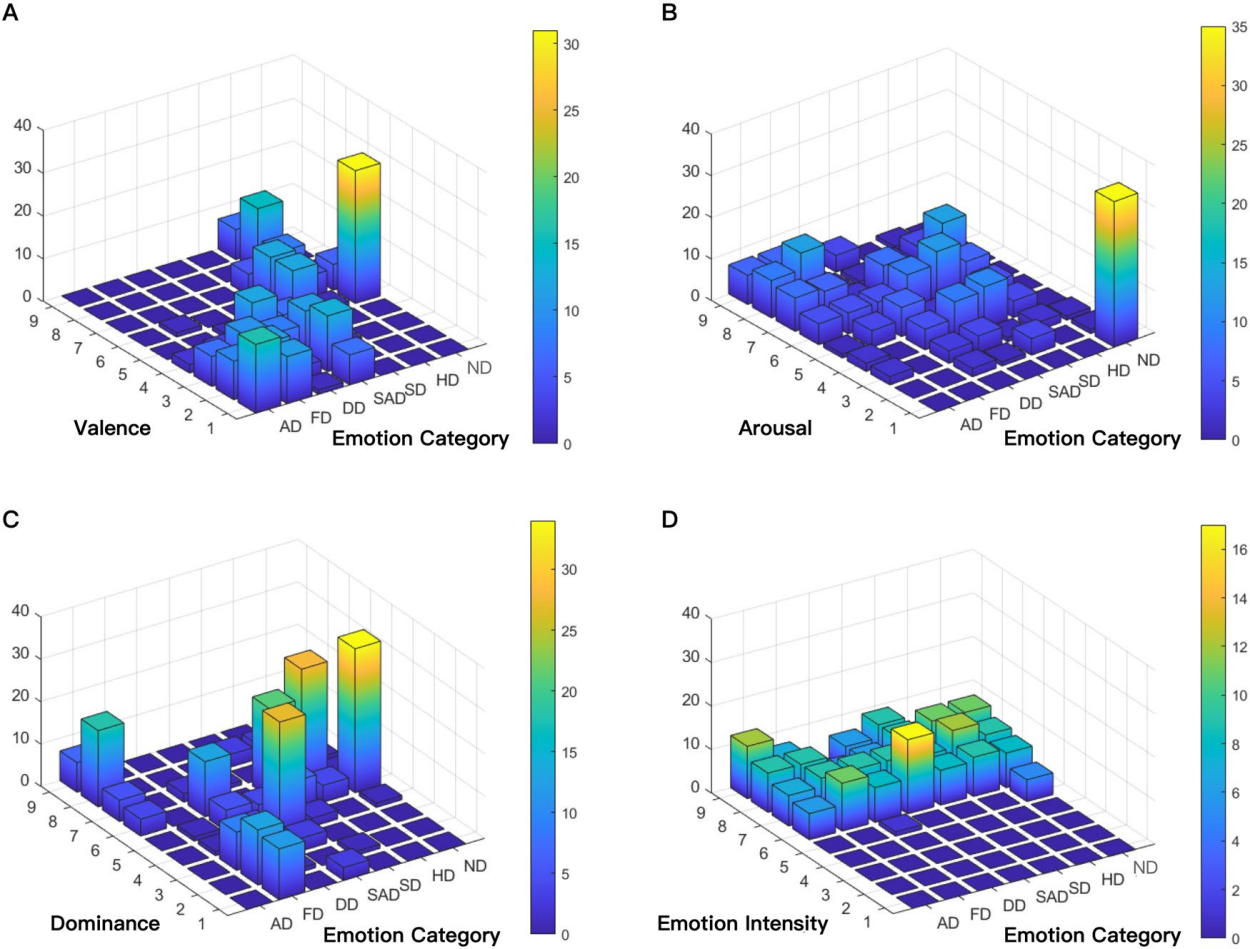
(**A**) The distribution of valence scores and emotion category, (**B**) The distribution of arousal scores and emotion category, (**C**) The distribution of dominance scores and emotion category, (**D**) The distribution of emotion intensity and emotion category.

The participants’ ratings scores of valence, arousal and dominance, emotion category and intensity were summarized in Fig. 7. The x-coordinate represents seven emotion category, the y-coordinate represents the values of the subjective scores, and the z-coordinate represents the total number of each item.

Figure 7(A) shows the distribution of valence. Valence means the positivity or negativity of an emotion according to the definition. Positive emotions have higher valence scores while negative emotions have lower valence scores. The data distribution shown in Fig. 7(A) conforms to this pattern. The valence scores of negative emotions (such as sad, angry, disgust and fear) are mainly located between 1 and 4 while the valence scores of positive or neutral emotions (such as neutral, happy and surprised) are mainly located between 5 and 9. By conforming to the regular distribution pattern, the valence score is verified.

Figure 7(B) shows the distribution of arousal. According to the definition, arousal ranges from excitement to relaxation. The data distribution shown in Fig. 7(B) consist with this pattern. As Fig. 7(B) shows, the arousal scores of neutral only located at the 1, which means the participants feel relaxed and have no positive or negative emotions at this moment. This feature meet our objective experience, which validate the reliability of arousal data.

Figure 7(C) shows the distribution of dominance. According to the valence-arousal-dominance model, dominance ranges from submissive to dominant. Fear is low-dominance and anger is high-dominance. The distribution of fear driving shown in Fig. 7(C) concentrated mainly between 1 and 3, which conforms to this pattern. The distributions of other emotions mainly located between 4 and 6, which shows that participants had a mid-level control towards these emotions. By conforming to the regular distribution pattern, the reliability of dominance score is verified.

Figure 7(D) shows the distribution of emotion intensity. Based on the definition of DES, the score of intensity and the strength of different emotions are positively related. The distribution verifies that participants’ emotions have been successfully stimulated since all of the data are distributed between 5 and 9 and most of them located between 6 and 9.

### Quality validation of physiological and behavioural data

**Fig. 8:**
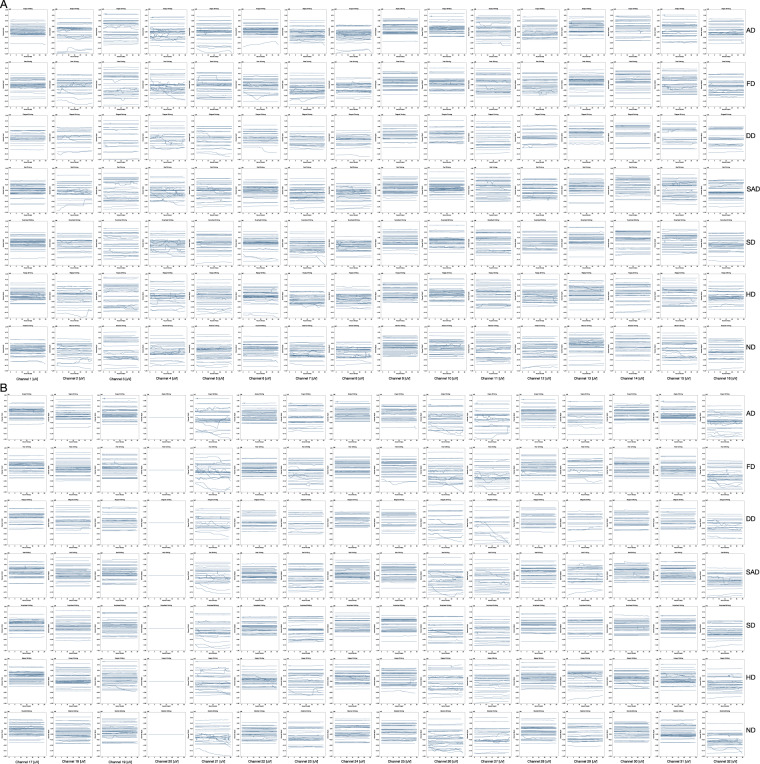
Time functions of EEG signals under different emotion states. Each row in the figure represents a different emotional state while driving(AD, FD, DD, SAD, SD, HD and ND). Each column of the figure represents signals obtained from different channels. (**A**) EEG signals of channel 1 to channel 16, (**B**) EEG signals of channel 17 to channel 32.

**Fig. 9:**
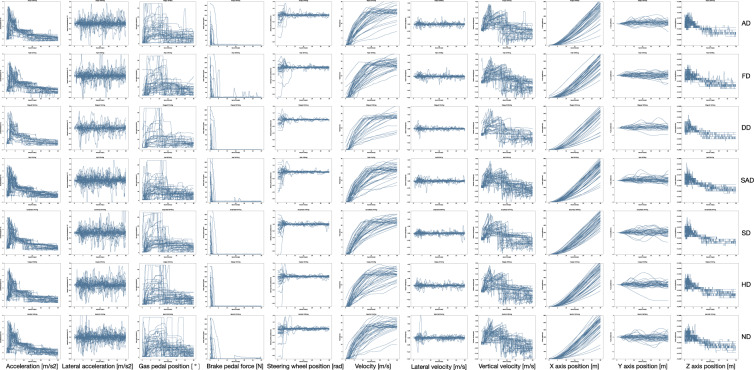
Time functions of driving behaviour under different emotion states. Each row in the figure represents a different emotional state while driving. From top to bottom, they are AD, FD, DD, SAD, SD, HD and ND. Each column of the figure represents a different dynamics signal.

For each variable involved in the research, we performed a visual display and a quality control, and the quality of data measurements has been thoroughly tested. The relevant signals were extracted as time function. The overall results per drive after quality control is shown in Figs. 8 and 9.

#### Physiological data

The device we used to obtain EEG signals is a head-mounted device. The data was obtained through the electrodes. Therefore, the quantity and length of participants’ hair will affect the contact of the electrodes as well as the results. Thus, good contact is necessary to get effective data. The valid data was extracted and shown in Fig. 8, which contained the trend curves of 32 channels of EEG signals under different emotional conditions. As we can see from Fig. 8, the signals collected by most channels have obvious similarities and trends except channel 20. The signals from channel 20 are invalid due to poor contact during the experiment.

#### Driving behavioural data

These variables include 11 dynamics parameters that represent driving behaviour, including accelerations, degree/force of gas/brake-pedal, velocities and positions, which provide a comprehensive description of driving behaviour. Figure 9 shows the time function of different driving behaviours under seven different emotions. Each sub-figure in Fig. 9 contains 40 curves and each curve represents the time functions of a participant’s driving signals. All of the driving behaviour data were acquired through a driving simulator mentioned above. As shown in Fig. 9, the tendency of driving behaviour data is consistent in the same emotion.

### Correlation analysis of physiology, behaviour and emotion labels

**Fig. 10:**
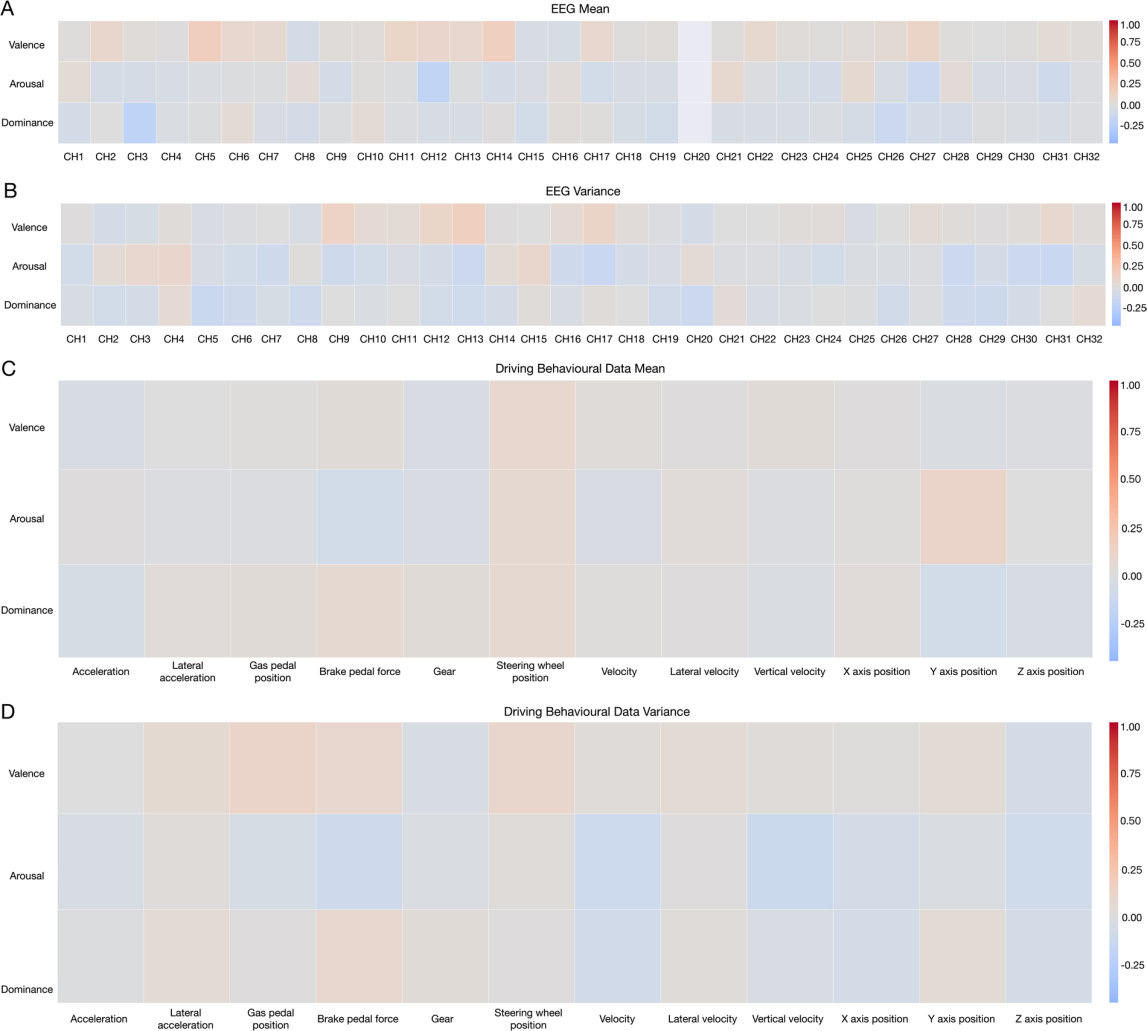
Correlation heatmap of signals. (**A**) Mean values of EEG signals and three dimensions scores of emotions, (**B**) Variance values of EEG signals and three dimensions scores of emotions, (**C**) Mean values of driving behavioural data and three dimensions scores of emotions, (**D**) Variance values of driving behavioural data and three dimensions scores of emotions.

Figure 10 shows the Spearman correlations analysis^44^ among 11 driving behaviours, 32-channel EEG signals and 3 dimensions of emotions. The correlations were shown in the form of heatmap. In Fig. 10, the data was first non-dimensionalized using the max-min normalization methods. Then, the mean and variance values of the data were calculated separately for each participant and the processed data was combined together with 3 dimensions of emotions scores. The Spearman’s correlations analysis was then used to obtain the correlation coefficients. The correlation coefficients are in a range of −1 to 1.

#### Physiological data and emotion labels

Fig. 10(A) shows the correlation heatmap between mean values of EEG signals and 3 dimensions scores of emotions. Correlations with high significance were noticed for the groups of valence-channel(CH)5/CH11/CH14/CH27, arousal-CH12/CH27/CH31 and dominance-CH3/CH26. Figure 10(B) shows the correlation heatmap between variance values of EEG signals and 3 dimensions scores of emotions. Correlations with high significance were noticed for the groups of valence-CH9/CH12/CH13/CH17, arousal-CH4/CH7/CH9/CH13/CH16/CH17/CH28/CH30/CH31 and dominance-CH5/CH6/CH8/CH20 /CH28/CH29.

#### Behavioural data and emotion labels

Fig. 10(C) shows the correlation heatmap between mean values of Driving behavioural data and 3 dimensions scores of emotions. The correlation coefficients were normalized before the process. Correlations with high significance were noticed for the group of arousal-brake pedal force. Figure 10(D) shows the correlation heatmap between variance values of Driving behavioural data and 3 dimensions scores of emotions. Correlations with high significance were noticed for the groups of valence-gas pedal degree, arousal-brake pedal force, arousal-vertical velocity/velocity and dominance-velocity.

## Usage Notes

The user can use any video playback tool (e.g., QuickTime Player) to open the.MP4 file. The user can use any spreadsheet or workbook software to open the.CSV file. The data can be directly imported into Python, Matlab and other statistical or programming tools for analysis. We recommend that users check the sample report in the database for further clarification.

### Potential applications

With the help of various data mining techniques, the dataset can be used for the analysis of the relationship between the emotion-physiology-behaviour-personality trait of human drivers^45,46^. It can be used to analyze the driving risks caused by the emotions of human drivers^13,47^. It can also be used to analyze the difference between human emotion expression in driving scenes and life scenes^6,48^. The dataset can also be used to analyze the cognitive and behaviour changes of human drivers in different emotions in the driving environment, and then conduct research on human drivers’ emotion regulation strategies^46,49^.

Moreover, by applying various machine learning techniques, based on the collected driving behaviour, EEG, facial expressions, driving posture, and road scene information, the dataset can be used to develop single-modal/multi-modal driver emotion monitoring algorithms^35,50^. Accurate and efficient emotion monitoring algorithms will help the emotion-aware interaction between human drivers and intelligent vehicles to improve driving safety and comfort, and increase human trust in machines^46,51^. Besides, The PPB-Emo dataset will also benefit human emotion research in other daily tasks.

### Limitations and future works

Driver emotion induction. This study used video-audio clips to induce driver emotions. Although all these video-audio clips have been validated to be effective in eliciting expected human driver emotions, this study cannot completely rule out the possibility that there may be difference between video-audio clips induced emotion and real on-road driving scenarios induced emotion. In the future, we will further conduct on-road driving experiments to study the emotions of human drivers in real on-road driving scenarios.

Participants.There was a gender imbalance in all three experiments in this study, with a 3:1 male to female ratio. Although this is basically the same as the male-to-female ratio of Chinese drivers^52^, this may affect the use of the dataset. Future research should maintain a balanced gender ratio as much as possible. In addition, in Experiment II, 409 Chinese participants were invited to answer the online questionnaires. Since the scenarios to elicit the same emotions might vary in different cultures^53^, the induction effect of the driving scenarios provided in Experiment II on different culture groups needs further research.

## Data Availability

The data pre-processing methods and procedures of validation mentioned in the technical validation section were carried out in Jupyter Notebook. Python version 3.5.8 was used throughout. The correlation analysis and distribution display are conducted using seaborn, sciki-learn^54^ and pandas packages. The codes and a brief description(readme.md) have been uploaded.
